# Death attitudes and good life experience: the mediation and suppression effects of intrinsic and extrinsic goals

**DOI:** 10.3389/fpsyt.2025.1567600

**Published:** 2025-04-25

**Authors:** Yuanyuan Wang, Fuhua Pei, Yisheng Yang, Junxiu Wang

**Affiliations:** ^1^ School of Psychology, Inner Mongolia Normal University, Hohhot, Inner Mongolia Autonomous Region, China; ^2^ School of Psychology, Qufu Normal University, Qufu, Shandong, China; ^3^ The Affiliated Kangning Hospital of Wenzhou Medical University, Wenzhou, Zhejiang, China; ^4^ School of Mental Health, Wenzhou Medical University, Wenzhou, Zhejiang, China

**Keywords:** death anxiety, neutral acceptance, intrinsic goals, extrinsic goals, good life experience

## Abstract

This study employs Heidegger's philosophy, Goal Content Theory, and Terror Management Theory to investigate the roles of intrinsic and extrinsic goals in the relationship between death attitudes (neutral acceptance vs. death anxiety) and the good life experience. Analyzing nationally representative data from the Chinese Social Mentality Survey (N=10,195), structural equation models revealed three key findings: (1) Neutral acceptance positively correlated with the good life experience, whereas death anxiety demonstrated negative associations; (2) In the primary conceptual model (Model 1), intrinsic goals mediated while extrinsic goals suppressed the relationship between the death attitudes and the good life experience; (3) Further model validation indicated that consistent suppression effects of extrinsic goals in national stability (Model 2) and personal richness (Model 4) frameworks, with distinctive dual mediation emerging in family happiness (Model 3). Notably, the personal richness model (Model 4) showed non-significant total effects despite preserved mediation patterns. These results advance existential psychology by elucidating culture-specific mechanisms through which death attitudes influences well-being in Chinese populations, while providing empirical validation and theoretical refinement of Goal Content Theory's cross-cultural applicability.

## Introduction

In the contemporary era, individuals are exposed to death-related information on an unprecedented scale and with unparalleled intensity. For example, as of March 5, 2023, a total of 1,405 conflict-related deaths were recorded in Ukraine (1). According to the WHO’s World Health Statistics 2023 (2), nearly 15 million excess deaths globally were attributed to COVID-19 during 2020 and 2021. Additionally, the 2023 Turkey-Syria earthquakes resulted in approximately 50,000 fatalities (3), among other significant events. Importantly, social media significantly amplifies this exposure through real-time dissemination, whether via viral infographics tracking pandemic mortality rates or livestreamed footage from conflict zones. Consequently, these streams of information make it increasingly challenging for individuals to suppress or evade the inevitable reality of death, along with the existential anxiety and uncertainty it entails (4).

### Death attitudes and good life experience

While biological death is an inevitable reality, attitudes toward it can be diverse, changeable and malleable. Death attitude can serve as a critical factor in shaping individuals’ lives (5). Zhong et al. categorized death attitudes into three types: disengaged, calm reflectors, and anxious reflectors. They found that anxious reflectors reported lower levels of well-being compared to the other two groups (6). Another research also found that death can diminish subjective vitality and self-regulatory capacity, primarily on individuals with low levels of interdependent self-construction (7). Jo and Lee’s research revealed that college students’ attitudes toward death are closely correlated with their levels of self-efficacy, depression, and life satisfaction (8).

The initial psychological research on death attitudes primarily focused on death anxiety (9). Death anxiety is the most direct and instinctive emotion and attitude of individuals toward death. And it refers to a psychological state of anxiety that arises intrinsically within an individual upon being reminded of the inevitability of death, reflecting the perceived threat posed by mortality (7). Some researchers have also attempted to broaden the scope of investigation to include positive attitudes toward death. By the mid-1980s, research on death attitudes had expanded to encompass a broader and more diverse range of perspectives, as exemplified by the work of Wong, Reker, and Gesser (10). They measured death attitudes in multiple dimensions, encompassing not only the negative aspects such as death anxiety or death escape, but also the positive aspect of neutral acceptance. Neutral acceptance refers to an attitude that views death as an integral and inescapable part of life, acknowledging it as one of the unchangeable realities of existence.

### Death attitudes and well-being

Previous research has demonstrated that the impact of death attitudes on well-being is different. Basharpoor et al. assert that elderly adults who hold positive attitudes toward death tend to perceive it as a path to eternal well-being, while negative attitudes toward death can impair elderly individuals’ quality of life (11). Some studies have shown that death anxiety is a common issue in patients and can impact their quality of life (12, 13). There are also studies indicating that death anxiety negatively affects harmonious passion (14, 15). During the 2019 coronavirus disease pandemic, a significant body of research focusing on death attitudes emerged. These studies revealed that death anxiety negatively impacts people’s lives (16), nurses’ satisfaction with life (17, 18), young people’s mental health (19), and psychological well-being (20). The current research mainly focuses on specific groups, such as the elderly, college students, patients and medical staff, with limited attention has been paid to the impact of death attitudes toward death on the well-being of the general public, especially in China. One important reason is since ancient times, the Chinese have generally been hesitant to directly address the topic of death, and view it as taboo (6). Although the major research on death anxiety focuses on specific groups, the results of all studies indicate that death anxiety has a negative impact on subjective well-being.

Meaning management theory (MMT) suggests that death can be used as a motivation to pursue a more meaningful life. Chang and Thimm (21) argues that death can be the basis for a more meaningful life and existence. Their research indicates that a good death mindset is associated with higher life satisfaction, fewer symptoms of depression, anxiety, and stress, as well as lower health anxiety. Research on neutral acceptance primarily focuses on the classification and typology of death attitude. The limited existing research has demonstrated that individuals who maintain positive attitudes toward death can enhance their well-being (8, 11). And other research claims that neutral acceptance toward death appears to be positively associated with higher levels of life satisfaction (9). So we believe that neutral acceptance of positive death attitudes positively predicts subjective well-being.

### Subjective well-being and good life experience

Subjective well-being (SWB) is a comprehensive psychological index used to measure individuals’ perceptions of their living conditions, which refers to an individual’s overall evaluation of their life quality based on personal standards (22). In recent years, relevant studies have primarily assessed subjective well-being through the indicators of life satisfaction, positive affect, and negative affect (23). Among them, life satisfaction, a classic measure of well-being, is utilized to assess the aspect of life quality related to overall life.

To gain a deeper understanding of Chinese people’s perceptions and evaluations of well-being, researchers have developed the Good Life Experience Questionnaire using methods such as word association, high-frequency word analysis and cluster analysis, and found that Chinese people’s understanding of well-being can be divided into three dimensions: personal richness, family happiness and nation stability (24). While the concept of a good life experience is divided into three levels: nation, family and individual, it is also categorized into three aspects: stability, happiness and material well-being. This aligns with traditional Chinese culture.

Family-nation sentiment is a cultural gene deeply embedded in the Chinese people’s heritage (25), and has been ingrained in both the conscious and unconscious minds of every Chinese individual. As stated in “Mencius • Li Lou (Part I)”: “The foundation of the world lies in the state, the foundation of the state lies in the family, and the foundation of the family lies in oneself.” (26) “Cultivating the self, harmonizing the household, governing the state, and pacifying all under Heaven,” is a Confucian classic maxim that has been passed down through the ages. Confucianism emphasizes self-cultivation, while also stressing family regulation, state governance and universal peace, highlighting the intricate interconnection between the individual, family and station. In Chinese, the term “国家” (guójiā) literally combines the characters for “station” and “family,” reflecting this deep-seated connection. In Chinese traditional culture, individual well-being is not solely derived from personal achievements and satisfaction but also from one’s contributions to the collective and the recognition received from it. Poll results indicate that family social connections, national development, and the overall well-being of Chinese people are closely intertwined (27). Researchers have identified a fundamental difference between Chinese and Western conceptions of happiness: the Chinese perspective emphasizes national strength, international standing, social environment, and familial harmony as primary determinants of individual well-being (27). These insights suggest that family harmony, social stability, and national development are crucial contributors to Chinese individual well-being (28).

When pursuing well-being, Chinese individuals not only focus on personal dimensions but also place significant expectations on their families and the nation. Mainstream research on well-being has traditionally focused on individual dimensions such as life satisfaction, vitality, and positive or negative emotions, often neglecting the dimensions of family and station. However, we argue that the family and national dimensions are vital sources of happiness. For instance, during times of war, individuals happiness is compromised; similarly, family breakdowns can adversely affect the happiness of each member. Therefore, this study adopts the good life experience as a measure of subjective well-being, examining it from the three dimensions of individual, family, and station more comprehensively.

In conclusion, Hypothesis 1 and Hypothesis 2 were formulated. Death anxiety, as a component of negative death attitudes, is expected to negatively predict the good life experience (H1). And neutral acceptance, a positive death attitude, is expected to positively predicts the good life experience (H2).

### The mediation effects of goal contents

In the Goal Content Theory within Self-Determination Theory (SDT), goal contents are categorized into intrinsic and extrinsic goals. Intrinsic goals mainly refer to self-growth and other goals closely related to basic psychological needs, such as autonomy and competence. Extrinsic goals mainly refer to wealth, status and other goals focusing on obtaining rewards and the positive evaluations of others (29). Goal Content Theory further claims that intrinsic and extrinsic goals have distinct effects on the subjective well-being. Research by Schmuck, Kasser, and Ryan (29, 30) demonstrated that intrinsic goals positively predicted well-being, whereas extrinsic goals are negatively associated with it.

### Death attitudes and goal contents

Heidegger (5) believes that when individuals confront death directly, they will return to their authentic selves and live more genuine lives; conversely, when people avoid death, they sink into everyday things and live lives that are in-authenticity. He further suggests that only through an authentic life can one discover the profound meaning of existence. Conversely, getting immersed in daily trivial pursuits leads to the erosion of one’s unique essence. Following this logic, self-growth and other intrinsic goals that can satisfy individuals’ psychological needs and enhance individual’s meaning in life are the important part of an authentic life. Pursuits such as status and wealth which make individuals more prone to being immersed in daily trifles are part of an inauthentic life. So we posit that varying death attitudes exert distinct influences on goal contents. Specially, a positive attitude toward death will enable people to lead more authentic goals, such as in intimate relationships and other inner goals; while a negative attitude toward death will make people live less self-consciously, and more inclined to do things related to extrinsic goals like social status.

The Terror Management Theory (TMT) posits that the core of human behavioral motivation lies in managing the anxiety of death. To alleviate this anxiety, individuals adopt different defense mechanisms, including both proximal and distal defenses. Building on the TMT, the Dual-Process Model (31) claims that when individuals are consciously aware of death-related thoughts, individuals will first engage in proximal defenses to decrease the death anxiety (4), which involve suppression or distraction by focusing attention on extrinsic and death-unrelated matters. Relevant studies have shown that death consciousness has a greater preference for high-status products (32). And death anxiety significantly predicts extrinsic goals such as wealth (33). Extrinsic goals can also play a role in diverting attention from death and can temporarily alleviate the death anxiety (29, 34). That is to say, when death elicits anxiety in individuals, they tend to place increased emphasis on extrinsic goals such as monetary wealth, social fame, and personal image.

Neutral acceptance of death refers to the conscious and rational recognition of the inevitable prospect of one’s own death (the cognitive component in the face of death) as well as the positive emotional evaluation of this recognition (the integration of death and the emotional component) (9, 10). So it is closely related to the continuous and in-depth exploration of mortality, reflecting individuals’ deeper understanding of death. People who hold neutral acceptance try to reduce their death anxiety by doing things that are more meaningful and thereby enhancing their life value. Empirical research has shown that when individuals engage in a deep and rational exploration of death, their goal orientation shifts from extrinsic to intrinsic goals (34, 35). Consequently, we posits that neutral acceptance is an important factor enhancing the intrinsic goals.

Individuals’ goal orientation is capable of undergoing a transition. Research indicates that when people focus too much on extrinsic goals, extrinsic goals may crowd out behavior that meets psychological basic needs, such as building connections and engaging in prosocial activities (36). As previously stated, when individuals engage in a profound and rational examination of death, their goal orientation transitions from extrinsic to intrinsic goals (35). These findings reflect a shift in goal orientation, either from intrinsic to extrinsic goals or from extrinsic to intrinsic goals (34). In summary, death anxiety tends to drive individuals toward prioritizing extrinsic goals, which may lead to a corresponding reduction in the pursuit of intrinsic goals. Conversely, a deep and rational reflection on death encourages the pursuit of intrinsic goals, potentially resulting in varying degrees of diminished attention to extrinsic goals.

### Goal contents and good life experience

In line with the Goal Content Theory, the positive relationship between intrinsic goals and well-being has been empirically validated across different countries (37, 38), including China, as demonstrated in studies such as Huang et al. (39) and Cai (40). While the correlation between these two variables has been found to be stable and consistent, this study primarily examines the different mediating effects of intrinsic and extrinsic goals in the mechanism of death attitudes on good life experience, so intrinsic goals are integrated into the model.

Different from the well-being effect of intrinsic goals, the generalizability of well-being effect of extrinsic goals remains to be empirically tested in other cultural contexts. Empirical evidence suggests that the pursuit of extrinsic aspirations is not always detrimental to well-being (41). A meta-analysis found universal wellness is compromised only when extrinsic goals predominate in the overall goal structure (42). AK-means cluster analysis of 835 Croatian university students identified four distinct goal orientation profiles. And revealed that the group with high scores in both intrinsic and extrinsic goals exhibited the highest levels of well-being, followed by the group with high intrinsic but low extrinsic goal scores (43). These findings suggest that both intrinsic and extrinsic goals can contribute positively to overall well-being. Moreover, a study conducted in Hungary indicated that intrinsic goals significantly predicted well-being, but extrinsic ones exhibited a weak yet positive association with well-being (44). Notably, cross-cultural comparisons demonstrate variability. A study comparing Chinese and North American samples observed a weak yet statistically significant positive association between the relative emphasis on extrinsic goals and well-being in Chinese samples. However, no significant correlation between these variables was found in North American samples (38). Additionally, similar correlation findings were observed in local studies in China (39, 45). Research has confirmed that if extrinsic goals can help people achieve basic economic security or intrinsic goals, then extrinsic goals will not reduce well-being (41). These findings collectively suggest that extrinsic goals are positively associated with well-being. In this study, the good life experience is used as an indicator to measure the subjective well-being of Chinese individuals. Therefore, we propose that extrinsic goals are positively related to good life experience among Chinese individuals.

In summary, death attitudes shape individuals’ goal orientation, and the goal contents, in turn, influence their good life experiences. Furthermore, when individuals primarily orient themselves toward a certain type of goal (either intrinsic or extrinsic goal), their pursuit of the alternative goal (either extrinsic or intrinsic goal) is likely to diminish correspondingly. Therefore, we propose that intrinsic and extrinsic goals may play different direction mediation effects between death attitudes and good life experience in opposite directions. Specifically, death anxiety negatively influences good life experience via intrinsic goals (H3); neutral acceptance positively impacts good life experience through intrinsic goals (H4); death anxiety positively affects good life experience via extrinsic goals (H5); and neutral acceptance negatively influences good life experience through intrinsic goals (H6).

**Figure 1:**
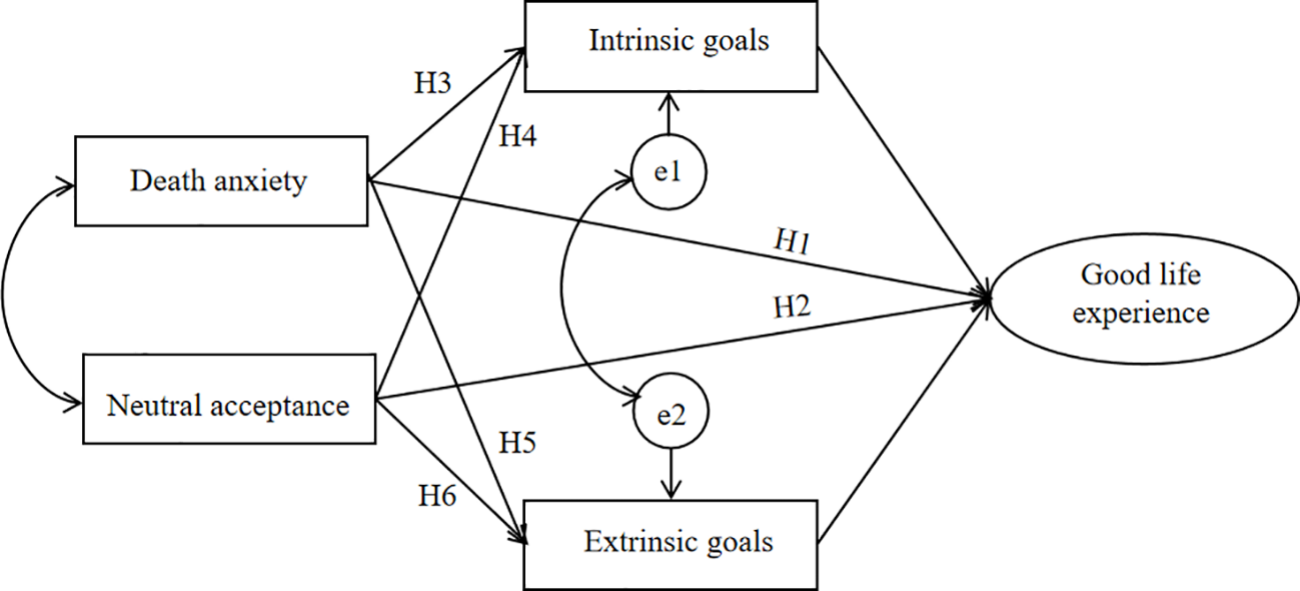
The hypothetical structure model.

Modern individuals encounter unparalleled exposure to mortality-related information, necessitating a direct confrontation with the inevitability of death while addressing the accompanying existential anxieties and uncertainties. However, throughout history, the Chinese have predominantly exhibited a reluctance to directly confront the topic of death, even treating it as a taboo, and the death attitudes related research remains few in China. Moreover, research conducted with Chinese participants examining the influence of extrinsic goals on well-being has yielded inconsistent findings. Finally, the perception of well-being differs significantly across cultures. While existing studies predominantly rely on Western scales or paradigms, this study employs the culturally grounded concept of good life experience as an indicator of well-being, reflecting a localized approach to well-being measurement. So based on the large-sample data, this study investigates the impact of death anxiety and neutral acceptance on good life experience and explores its underlying mechanisms in Chinese individuals. The hypothesized model is presented in **Figure 1**.

## Materials and methods

### Data source and sampling procedure

The data for this study were obtained from a nationwide serial survey—the Chinese Social Mentality Survey (CSMS)—conducted by the Chinese Academy of Social Sciences(CASS). The survey was administered through the “Questionnaire Treasure” APP, which distributed questionnaires to approximately 1.1 million users across 346 prefecture-level cities nationwide from September 11, 2020 to April 29, 2021. Snowball sampling was subsequently employed to expand the sampling range. Ultimately, 10,195 individuals voluntarily participated in the survey. Respondents were drawn from all 31 provincial administrative regions, including Beijing Municipality, Shandong Province, and the Inner Mongolia Autonomous Region, among others. Detailed demographic information of the participants is provided in **Table 1**.

**Table 1:** Participant’s demographic profile.

Particulars	Description	Percentage (%)/Mean
Gender	Male	43.3
	Female	56.7
Age	41.61	
Education	Never attended school	1.5
	Primary school	11.8
	Junior high school	29.6
	Senior high school	22.9
	Technical secondary school	5.7
	Vocational high school/ technical school	1.7
	Junior college	14.5
	Bachelor’s degree	11.8
	Postgraduate (including current student)	0.4
Personal income	3000 yuan and below	46
	3000-7000 yuan	44.9
	7000-15000 yuan	7.4
	Over 15000 yuan	1.8
Religious belief	Yes	8.5
	No	91.5

### Measurement scales

#### Death Attitudes Profile-Revised (DAP-R)

The neutral acceptance and death anxiety were measured by the DAP-R adapted from Tang et al. (46) and originally developed by Wong, Reker, and Gesser (10), employs a seven-point Likert scale where 1 signifies strongly disagree and 7 signifies strongly agree. Specifically, death anxiety was assessed using the item “The prospects of my own death arouses anxiety in me”. Neutral acceptance was measured by two items: “Death is a natural aspect of life” and “Death should be viewed as a natural, undeniable, and unavoidable event”. The Cronbach’s α for the neutral acceptance was 0.89.

#### Aspiration Index

The Aspiration Index was developed by Kasser and Ryan (47) and later modified for the Chinese context by Tang et al. (48). Consisting of 35 items, scored on a seven-point Likert scale with responses ranging from “very unimportant” to “very important”. It contains two parts of intrinsic and external goals. The intrinsic goals are categorized into four dimensions: affiliation (e.g., having good friends that I can count on), community feeling (e.g., contributing to the improvement of others’ lives), self-acceptance (e.g., knowing and accepting one’s true self) and physical fitness(e.g., maintaining physical health). Extrinsic goals were divided into three dimensions: financial success (e.g., to be a very wealthy person), appealing appearance (e.g., staying current with fashions in hair and clothing) and social recognition (e.g., being admired by many people).

Some researchers have expanded on these dimensions, adding self-transcendence and physical self. They have demonstrated that community feeling can serve as both an intrinsic and a self-transcendence goal, physical fitness can function as both an intrinsic and a physical self goal, and financial success is considered both an extrinsic and a physical self goal. As this study focuses on the influence of intrinsic and extrinsic goals, pure single-dimensional affiliation and self-acceptance were taken as items to measure pure intrinsic goals. Items that measured appealing appearance and social recognition were taken as purely extrinsic goals. The Cronbach’s α for both intrinsic and extrinsic goals was 0.92.

#### Satisfaction With Life Scale (SWLS)

The Satisfaction With Life Scale consists of five items, and was originally developed by Diener, Emmons, Larsen, and Griffin (49). The scale is scored on a seven-point Likert scale ranging from “strongly disagree” to “strongly agree”. The five items in the scale are respectively as follows: (a) In most ways my life is close to my ideal. (b) The conditions of my life are excellent. (c) I am satisfied with my life. (d) So far I have gotten the important things I want in life. (e) If I could live my life over, I would change almost nothing. The Cronbach’s α for this scale was 0.88.

#### The Good Life Experience Questionnaire

The 18-item Good Life Experience questionnaire was developed by the Chinese Academy of Social Sciences(CASS) to measure participants’ experience of a good life. The questionnaire was divided into three dimensions: (a)National stability and order (items include “I feel society is stable”), (b)family happiness (items include “I feel my home life is very sweet”) and personal richness (items include “I have the money to do what I want”). Participants gave responses on a seven-point Likert scale from 1 (strongly disagree) to 7 (strongly agree). Higher average scores indicate a higher level of good life experience. The total Cronbach’s α for the questionnaire was 0.92, with Cronbach’s α values for the three sub-scales being 0.90, 0.82, and 0.83 respectively.

### Statistical analysis

Descriptive statistics and related analyses were conducted using SPSS 27.0. The hypothesized structural equation models were tested using AMOS 26.0.

## Results

### Common method bias test

The Harman’s single-factor test was conducted to test the common method bias of the used items in the self-reported scales of DAP-R, AI, SWLS, and the Good Life Experience questionnaire. The results showed that 12 factors had eigenvalues greater than 1, and the largest factor explaining only 26.95% of the variance, which was below the critical threshold of 40%. The findings indicate that there is no significant common method bias in the current study.

### Correlation analysis

**Table 2** reports the means, standard deviations, and Pearson correlations. The correlation analysis revealed that death anxiety was negatively associated with nation stability, family happiness and good life experience (*ps* < 0.01), and positively with the extrinsic goal (*ps* < 0.01). In contrast, neutral acceptance exhibited opposite patterns: it was positively associated with the nation stability, family happiness and good life experience (*ps* < 0.01), and negatively with the extrinsic goal. In addition, the neutral acceptance was positively related to intrinsic goals (*ps* < 0.01). These findings indicate that the two death attitudes exhibit opposite correlations with the mediating variables of intrinsic and extrinsic goals, and dependent variables such as nation stability, family happiness, and the good life experience, offering preliminary evidence in favor of the proposed research hypotheses and lending support to further hypothesis testing.

**Table 2:** Descriptive statistics and correlation analysis for all variables.

Variables	M	SD	1	2	3	4	5	6	7
1 Death anxiety	3.52	1.54	–						
2 Neutral acceptance	5.21	1.40	0.22^**^	–					
3 Intrinsic goal	5.29	0.96	-0.001	0.24^**^	–				
4 Extrinsic goal	4.58	1.08	0.07^**^	-0.05^**^	0.59^**^	–			
5 Nation stability	5.64	0.80	-0.05^**^	0.07^**^	0.26^**^	0.18^**^	–		
6 Family happiness	5.65	0.89	-0.04^**^	0.09^**^	0.29^**^	0.18^**^	0.67^**^	–	
7 Personal richness	5.06	1.02	0.001	-0.01	0.19^**^	0.28^**^	0.62^**^	0.57^**^	–
8 Good life experience	5.48	0.77	-0.04^**^	0.06^**^	0.28^**^	0.24^**^	0.91^**^	0.84^**^	0.84^**^

*p < 0.05, **p < 0.01, ***p < 0.001.

Neither death anxiety nor neutral acceptance was significantly correlated with life satisfaction (*ps* > 0.05) and personal richness. Furthermore, both intrinsic and extrinsic goals were positively correlated with the life satisfaction, overall good life experience and its two dimensions of nation stability and family happiness (*ps* < 0.01). Life satisfaction, good life experience and its two dimensions were also positively correlated with each other (*ps* < 0.01). Despite the significant and positive correlations among various outcomes, distinct patterns are evident in the relationships between death attitudes and life satisfaction compared to the good life experience. Specifically, no statistically significant association was identified between death attitudes and life satisfaction or personal richness. In contrast, a significant positive correlation was observed between death attitudes and the good life experience.

### Hypothesis model testing 1: good life experience as an outcome (Model 1)

The mediation analysis was conducted to examine the mediating role of intrinsic and extrinsic goals in the relationships between death attitudes and subjective well-being, including good life experience and life satisfaction. The results presented in **Table 3** indicated that the model-data fit for Model 1 was acceptable.

**Table 3:** Model fit indices for models.

Model Description	TLI	CFI	RMSEA
Model1 Good life experience as an outcome	0.96	0.99	0.06
Model2 National stability as an Outcome	0.99	0.99	0.03
Model3 Family happiness as an Outcome	0.99	0.99	0.03
Model4 Personal richness as an Outcome	0.97	0.98	0.05

**Figure 2:**
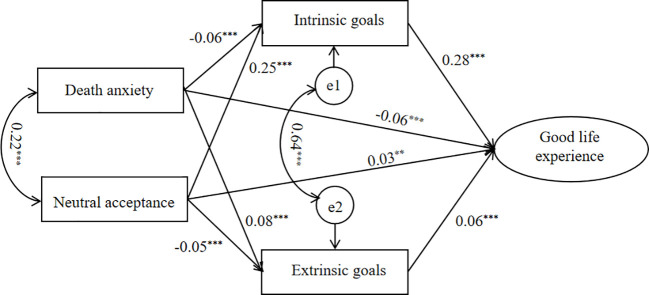
The model of good life experience as the outcome (Model 1). *p < 0.05, **p < 0.01, ***p < 0.001.

As shown in **Figure 2**, the direct effects of death anxiety (*β* = -0.06, *p* < 0.001, 95% *CI* = [-0.08, -0.03]) and neutral acceptance (*β* = 0.03, *p* < 0.05, 95% *CI* = [0.002, 0.05]) on the good life experience were significant. Death anxiety was negatively associated with the intrinsic goals (*β* = -0.06, *p* < 0.001, 95% *CI* = [-0.08, -0.04]) and positively associated with the extrinsic goals (*β* = 0.08, *p* < 0.001, 95% *CI* = [0.06, 0.10]). Conversely, neutral acceptance of death was positively associated with the intrinsic goals (*β* = 0.25, *p* < 0.001, 95% *CI* = [0.23, 0.28]) and negatively associated with the extrinsic goals (*β* = -0.05, *p* < 0.001, 95% *CI* = [-0.07, -0.03]). Finally, intrinsic goals and extrinsic goals both had positive effects on the good life experience (*β* = 0.28, *p* < 0.001, 95% *CI* = [0.25, 0.31]; *β* = 0.06, *p* < 0.001, 95% *CI* = [0.03, 0.09]).

As shown in **Table 4**, the total effect of death anxiety on the good life experience was significant (*β* = -0.07, *p* < 0.001, 95% *CI* = [-0.09, -0.05]). Overall, death anxiety exhibited a negative association with the good life experience. Furthermore, the indirect effect of death anxiety on the good life experience through intrinsic goals was significant (*β* = -0.02, *p* < 0.001, 95% *CI* = [-0.01, -0.005]). And the direct effect of death anxiety on the good life experience were significant. So the intrinsic goals played a partial mediation role in the relationship between death anxiety and the good life experience. The indirect effect through extrinsic goals was also significantly significant (*β* = 0.01, *p* < 0.001, 95% *CI* = [0.001, 0.003]). However, the path coefficient of the indirect effect (0.08 * 0.06) exhibited an opposite sign compared to the direct effect value (-0.06), indicating that extrinsic goals had a suppression effect between death anxiety and the good life experience (50). The suppression effect accounts (51) for 8% (|0.0048/-0.06| = 8%). The results show that, death anxiety negatively affects the good life experience, intrinsic goals play a mediation role, and extrinsic goals exert a suppression effect.

**Table 4:** Analysis of the mediation effects of intrinsic and extrinsic goals (Model 1).

Independent variable	Path	Coefficient	95% *CI* [LL, UL]	Effect proportion (%)	*P*
Death anxiety	Total effect of death anxiety	-0.07	[-0.09, -0.05]	–	0.001
	Death anxiety→good life experience	-0.06	[-0.08, -0.03]	83.33	0.001
	Total indirect effect of death anxiety	-0.01	[-0.02, -0.004]	16.67	0.002
	Death anxiety→intrinsic goals→good life experience	-0.017	[-0.01, -0.005]	23.33	0.001
	Death anxiety→extrinsic goals→good life experience	0.005	[0.001, 0.003]	8.00	0.000
Neutral acceptance	Total effect of neutral acceptance	0.09	[0.07, 0.12]	–	0.001
	Neutral acceptance→good life experience	0.03	[0.002, 0.05]	30.90	0.037
	Total indirect effect of neutral acceptance	0.07	[0.06, 0.08]	69.10	0.001
	Neutral acceptance→intrinsic goals→good life experience	0.07	[0.03, 0.04]	72.16	0.001
	Neutral acceptance→extrinsic goals→good life experience	-0.003	[-0.002, -0.001]	10.00	0.001

Neutral acceptance demonstrated a positive and significant total effect on the good life experience (*β* = 0.09, *p* < 0.01, 95% *CI* = [0.07, 0.12]). The indirect effect of influencing the good life experience through intrinsic goals was significant(*β* = 0.07, *p* < 0.01, 95% *CI* = [0.03, 0.04]). The direct effects of neutral acceptance on the good life experience were significant. These results indicated that intrinsic goals played a partial mediating role. The indirect effect of neutral acceptance through extrinsic goals was significant(*β* = -0.003, *p* < 0.01, 95% *CI* = [-0.002, -0.001]), but the path coefficient of the indirect effect (-0.05 * 0.06) is opposite in sign to the direct effect value (0.03), indicating that extrinsic goals have a suppression effect between neutral acceptance and the good life experience. The proportion of the effect size of the suppression effect is: |-0.005 * 0.06/0.03| = 10%. The results show that, neutral acceptance positively influences the good life experience, intrinsic goals have a mediation effect, but extrinsic goals play a suppression role.

### Hypothesis model testing 2: three dimensions of the good life experience as outcomes (Model 2 through 4)

**Figure 3:**
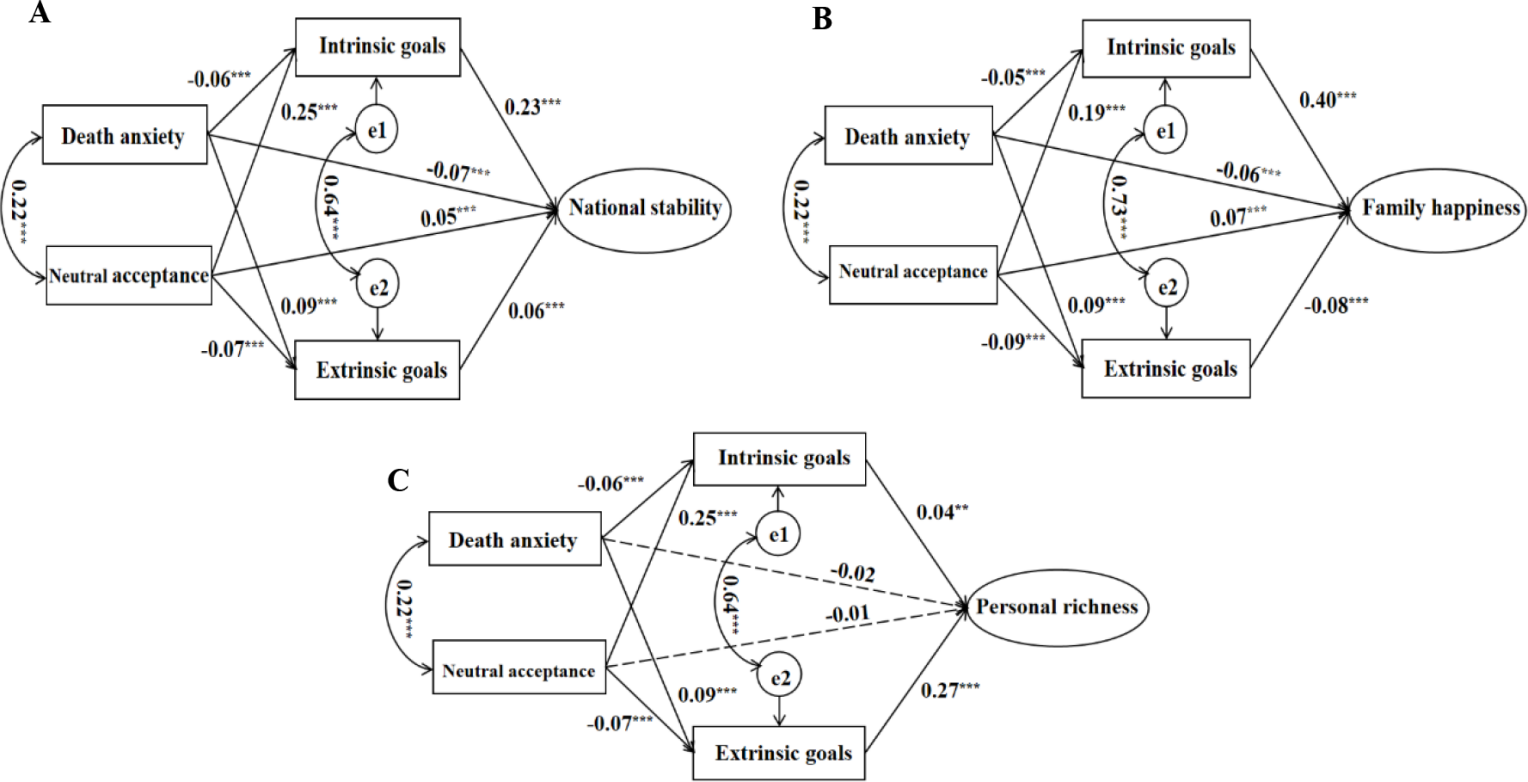
Three dimensions of the good life experience as respective outcomes (Model 2-4). *p < 0.05, **p < 0.01, ***p < 0.001.

To further explore the influence mechanism of the good life experience, and investigate whether death attitudes and goal contents differential influence the three dimensions of the good life experience, namely, national stability, family happiness, and personal richness, we further integrated these dimensions into separate mediation models for analysis. Model 2 (**Figure 3A**) investigate the mediating effects of intrinsic and extrinsic goals on the relationship between death attitudes and national stability. Model 3 (**Figure 3B**) serves as a mediation model with family happiness as the outcome variable, while Model 4 (**Figure 3C**) functions as a mediation model with personal richness as the outcome variable. As presented in **Table 3**, Models 2 through 4 showed adequate model-data fit.

In Model 2 (**Figure 3A** and **Table 5**), the total effect of death anxiety on national stability was statistically significant (*β* = 0.08, *p* < 0.001, 95% *CI* = [-0.10, -0.06]). The direct effect of death anxiety on national stability was significant (*β* = -0.07, *p* < 0.001, 95% *CI* = [-0.09, -0.05]). And the indirect effect of death anxiety on national stability through intrinsic goals was significant (*β* = -0.01, *p* < 0.001, 95% *CI* = [-0.01, -0.004]). The results indicated that intrinsic goals played a partial mediating role. Meanwhile, the indirect effect of death anxiety through extrinsic goals was significant(*β* = 0.01, *p* < 0.001, 95% *CI* = [0.002, 0.004]), but the path coefficient of the indirect effect (0.09 * 0.06) was opposite in sign to the direct effect value (-0.07), indicating that extrinsic goals have a suppression effect between neutral acceptance and national stability. The proportion of the effect size of the suppression effect was: |0.09 * 0.06/-0.07| = 7.71%. The results show that, neutral acceptance positively influences the national stability, intrinsic goals have a mediation effect, but extrinsic goals play a suppression role, which are consistent with Model 1.

On the other hand, the total effect of neutral acceptance on national stability was statistically significant (*β* = 0.10, *p* < 0.001, 95% *CI* = [0.08, 0.12]). The direct effect of neutral acceptance on national stability was significant (*β* = 0.05, *p* < 0.001, 95% *CI* = [0.02, 0.07]). And the indirect effect of neutral acceptance on national stability through intrinsic goals was significant (*β* = 0.06, *p* < 0.001, 95% *CI* = [0.03, 0.04]). The results indicated that intrinsic goals played a partial mediating role. Meanwhile, the indirect effect of neutral acceptance through extrinsic goals was significant(*β* = -0.004, *p* < 0.001, 95% *CI* = [-0.004, -0.001]), but the path coefficient of the indirect effect (-0.07 * 0.06) was opposite in sign to the direct effect value (0.05), indicating that extrinsic goals had a suppression effect between neutral acceptance and national stability. The proportion of the effect size of the suppression effect was: |-0.07 * 0.06/0.05| = 8.40%. The results show that, neutral acceptance positively influences the national stability, intrinsic goals have a mediation effect, but extrinsic goals play a suppression role, which are consistent with Model 1.

In Model 3 (**Figure 3B** and **Table 5**), the total effect of death anxiety on family happiness was significant (*β* = -0.09, *p* < 0.001, 95% *CI* = [-0.11, -0.07]). Overall, death anxiety exhibited a negative association with the family happiness. Furthermore, the indirect effect of death anxiety on family happiness through intrinsic goals was significant (*β* = -0.02, *p* < 0.001, 95% *CI* = [-0.01, -0.005]). Another indirect effect was through extrinsic goals which is also significant (*β* = -0.01, *p* < 0.001, 95% *CI* = [-0.005, -0.002]). And the direct effect of death anxiety on family happiness (*β* = -0.06, *p* < 0.001, 95% *CI* = [-0.08, -0.04]) was significant. Thus, both intrinsic and extrinsic goals partially mediate the relationship between death anxiety and family happiness.

The total effect of neutral acceptance on family happiness was significant (*β* = 0.15, *p* < 0.001, 95% *CI* = [0.13, 0.18]). The indirect effect of neutral acceptance on family happiness through intrinsic goals was significant (*β* = 0.08, *p* < 0.001, 95% *CI* = [0.04, 0.05]). Another indirect effect was through extrinsic goals which was also significant (*β* = 0.007, *p* < 0.001, 95% *CI* = [0.002, 0.01]). And the direct effect of neutral acceptance on family happiness (*β* = 0.07, *p* < 0.001, 95% *CI* = [0.04, 0.09]) was significant. Thus, both intrinsic and extrinsic goals partially mediate the relationship between neutral acceptance and family happiness. The results of Model 3 demonstrate that intrinsic and extrinsic goals function as parallel mediators.

In Model 4 (**Figure 3C** and **Table 5**), neither the total effect of death anxiety (*β* = 0.002, *p* >0.05, 95% *CI* = [-0.02, 0.03]) nor that of neutral acceptance (*β* = -0.02, *p* > 0.05, 95% *CI* = [-0.04, 0.01]) on personal richness was statistically significant. To investigate the reason for this lack of significance (51), further analysis indicated that the indirect effects of death anxiety on personal richness through intrinsic goals (*β* = -0.002, *p* < 0.001, 95% *CI* = [-0.003, -0.001]) and extrinsic goals (*β* = 0.02, *p* < 0.001, 95% *CI* = [0.01, 0.02]) were both statistically significant. Specifically, intrinsic goals acted as a mediator, while extrinsic goals exhibited a suppression effect. Similarly, neutral acceptance significantly influenced personal richness indirectly through intrinsic goals (*β* = 0.01, *p* < 0.001, 95% *CI* = [0.003, 0.01]) and extrinsic goals (*β* = -0.02, *p* < 0.001, 95% *CI* = [-0.02, -0.01). In this case, intrinsic goals demonstrated a suppression effect, whereas extrinsic goals served as a mediator. Notably, for the total effect of death anxiety, the proportions of intrinsic and extrinsic goals were 126.32% and 121.50% respectively, with their opposing effects canceling each other out, thereby negating the overall influence of death anxiety on personal richness. For neutral acceptance, the effect of proportions of intrinsic and extrinsic goals were both 100%, and their opposing effects precisely counterbalanced each other, resulting in no significant influence of neutral acceptance on personal richness.

**Table 5:** Analysis of the mediation effects in Model 2 through 4.

Independent variable	Path	Coefficient	95% *CI* [LL, UL]	Effect proportion(%)	*P*
**National stability (Model 2)**	**Total effect of death anxiety**	-0.08	[-0.10, -0.06]	–	0.001
	Death anxiety→national stability	-0.07	[-0.09, -0.05]	89.29	0.001
	Total indirect effect of death anxiety	-0.01	[-0.01, -0.001]	10.71	0.03
	Death anxiety→intrinsic goals→national stability	-0.01	[-0.01, -0.004]	17.60	0.001
	Death anxiety→extrinsic goals→national stability	0.01	[0.002, 0.004]	7.71	0.000
	**Total effect of neutral acceptance**	0.10	[0.08, 0.12]	–	0.001
	Neutral acceptance→national stability	0.05	[0.02, 0.07]	48.40	0.001
	Total indirect effect of neutral acceptance	0.05	[0.04, 0.06]	51.60	0.001
	Neutral acceptance→intrinsic goals→national stability	0.06	[0.03, 0.04]	55.66	0.001
	Neutral acceptance→extrinsic goals→national stability	-0.004	[-0.004, -0.001]	8.40	0.001
**Family happiness** **(Model 3)**	**Total effect of death anxiety**	-0.09	[-0.11, -0.07]	–	0.001
	Death anxiety→family happiness	-0.06	[-0.08, -0.04]	68.81	0.001
	Total indirect effect of death anxiety	-0.03	[-0.03, -0.02]	31.19	0.001
	Death anxiety→intrinsic goals→family happiness	-0.02	[-0.01, -0.005]	22.94	0.001
	Death anxiety→extrinsic goals→family happiness	-0.01	[-0.01, -0.002]	8.26	0.001
	**Total effect of neutral acceptance**	0.15	[0.13, 0.18]	–	0.001
	Neutral acceptance→family happiness	0.07	[0.04, 0.09]	45.69	0.001
	Total indirect effect of neutral acceptance	0.08	[0.07, 0.10]	54.31	0.001
	Neutral acceptance→intrinsic goals→family happiness	0.08	[0.04, 0.05]	49.61	0.001
	Neutral acceptance→extrinsic goals→family happiness	0.01	[0.002, 0.01	4.70	0.001
**Personal richness** **(Model 4)**	**Total effect of death anxiety**	0.002	[-0.02, 0.03]	–	0.42
	Death anxiety→personal richness	-0.02	[-0.04, 0.003]	–	0.03
	Total indirect effect of death anxiety	0.02	[0.02, 0.03]	–	0.001
	Death anxiety→intrinsic goals→personal richness	-0.002	[-0.003, -0.001]	126.32	0.001
	Death anxiety→extrinsic goals→personal richness	0.02	[0.01, 0.02]	121.50	0.000
	**Total effect of neutral acceptance**	-0.02	[-0.04, 0.01]	–	0.64
	Neutral acceptance→personal richness	-0.01	[-0.03, 0.02]	–	0.32
	Total indirect effect of neutral acceptance	-0.01	[-0.02, 0.001]	–	0.30
	Neutral acceptance→intrinsic goals→personal richness	0.01	[0.003, 0.01]	100	0.001
	Neutral acceptance→extrinsic goals→personal richness	-0.02	[-0.02, -0.01]	100	0.001

The bold text denotes the total effects of neutral acceptance and death anxiety across 3 models, serving to differentiate these from direct and indirect effects.

## Discussion

This study examined the effects of death attitudes on good life experience among the general public in China, as well as the mediating and suppressive roles of intrinsic and extrinsic goals. To achieve this, four models were developed. In Model 1, the outcome variable was the good life experience, whereas in Models 2, 3, and 4, the dependent variables were national stability, family happiness, and personal richness, respectively. The findings of Model 1 revealed that death anxiety negatively predicted the good life experience, while neutral acceptance positively predicted it, and the intrinsic goal acted as a mediator in this relationship, whereas the extrinsic goal functioned as a suppressor. Furthermore, the model results examining the impacts of death attitudes on the three dimensions of good life experience revealed that, in Models 2 and 3, death anxiety negatively predicted the good life experience, whereas neutral acceptance positively predicted it. These findings are consistent with the results of Model 1. Additionally, in Models 2 and 4, the intrinsic goal served as a mediator in this relationship, while the extrinsic goal acted as a suppressor, consistent with Model 1. But in Model 3, both intrinsic and extrinsic goals exhibited mediating effects. Notably, in Model 4, neither the total effect nor the direct effect of death attitudes on the good life experience reached statistical significance. The first reason is likely because personal richness primarily reflects in one’s economic status, which is an objective reality and is less susceptible to significant influence by one’s death attitudes. The second reason is that the mediation effect of the intrinsic goals and the suppression effect of the extrinsic goals counterbalanced each other, although the indirect effects were significant.

### Death attitudes and good life experience

Based on the results of Model1 through 3, death anxiety negatively predicted the good life experience, while neutral acceptance of death positively predicted it. Specifically, individuals experiencing death anxiety exhibit a significantly diminished effect on the good life experience; conversely, those who can accept and rationally understand death show a markedly enhanced effect. These findings are consistent with previous studies on subjective well-being (11, 13, 15, 18), thereby further expanding the research achievements in this field from the perspective of Chinese people’s good life experience. They also support the Meaning Management Theory and Chang and Thimm’s assertion that death attitudes not only have a negative impact on life, but also have a positive impact (21). More importantly, the results indicated that death attitudes are an important factor influencing the good life experience for the general public in China, and improving individual’s attitude toward death can enhance their quality of life experience.

### The mediation effect of intrinsic goals

Integrating the results of Models 1, 2, and 4, intrinsic goals mediate the relationship between death attitudes and good life experience, as well as its two dimensions: national stability and personal richness. Specifically, neutral acceptance positively influences good life experience and its two dimensions through the mediation of intrinsic goals, whereas death anxiety negatively impacts the outcomes via the same way.

When individuals adopt a neutral acceptance attitude toward death, they tend to prioritize intrinsic goals while placing less emphasis on extrinsic goals. In contrast, individuals experiencing death anxiety show a significantly reduced focus on intrinsic goals and a corresponding increase in the pursuit of extrinsic goals. These findings support Heidegger’s philosophical perspective, TMT and Goal Content Theory. The results indicate that death attitudes shape goal contents, which is consistent with previous research findings (32, 33) and further illustrate the shift between intrinsic and extrinsic goals (34, 35). The results that intrinsic goals positively predicted good life experience and its two dimensions, is consistent with the results of previous studies (37, 39, 40), and further verifies the Goal Content Theory that intrinsic goals can promoting subjective well-being.

### The suppression effect of extrinsic goals

Extrinsic goals exert a suppressive effect on the relationship between death attitudes and good life experience, as well as its two dimensions: national stability and personal richness, which contrasts with the findings related to intrinsic goals. Specifically, neutral acceptance exerts a negative impact on good life experience and its two dimensions through the extrinsic goals, whereas death anxiety had a positive influence on the outcomes via the same pathway.

Neutral acceptance was negatively correlated with extrinsic goals, whereas death anxiety exhibited a positive association with extrinsic goals. These findings further support Heidegger’s philosophical perspective and the Goal Content Theory. Specifically, individuals who experience death anxiety tend to prioritize extrinsic goals over intrinsic ones. The results also pertaining to death anxiety are consistent with Terror Management Theory, which posits that people compensate for their existential anxieties by pursuing extrinsic goals.

Extrinsic goals were positively associated with enhanced good life experience and its two dimensions. Although this finding is consistent with the study hypotheses, it is inconsistent with Self-Determination Theory. According to SDT, extrinsic goals can impair well-being by frustrating individuals’ basic psychological needs for autonomy, competence, and relatedness (47). The pursuit of extrinsic goals frequently involves ongoing social comparisons, resulting in feelings of exhaustion and powerlessness that undermine basic psychological needs. These negative effects may counteract the positive outcomes of achieving basic necessities such as shelter, safety, and warmth (42).

However, this study found that extrinsic goals positively predict the good life experience, although their impact is less pronounced compared to intrinsic goals, consistent with prior research (38, 39, 41, 44, 45). This suggests that the pattern wherein extrinsic goals undermine well-being does not fully hold in the Chinese context. We attribute this phenomenon to three main factors. First, Chinese culture is collectivist, where extrinsic goals such as “achieving social recognition” are particularly salient and important for individuals (41). Collectivist culture has been shown to enhance social adaptation by reducing conflict and negative emotions while fostering close relationships, thereby contributing to individual well-being (52). Second, extrinsic goals serve as indicators of personal competence. The achievement of extrinsic goals results in the satisfaction of the basic psychological need for competence. Spasovski (52) argues that in transitional societies with rapidly changing values, financial success and other extrinsic achievements become key indicators of personal ability, positively influencing subjective well-being, albeit potentially inefficiently and in the short term. Over the past few decades, China has experienced rapid economic development, leading to significant changes in societal values. Material possessions such as housing, cars, and money have become symbols of a successful life and personal competence, meeting basic psychological needs to some extent. So extrinsic goals can enhance the well-being of Chinese individuals. Third, extrinsic goals serve as essential means to achieve intrinsic goals. Studies indicate that in less affluent countries, people have fewer assets, making financial success and other extrinsic goals more closely tied to basic survival (41). Extrinsic goals facilitate the attainment of intrinsic goals (37) or higher-level aspirations. Although China has recently achieved poverty alleviation, the general public still possess relatively fewer extrinsic assets compared to developed countries. Achieving extrinsic goals can provide better living conditions for families and ensure improved learning opportunities. These factors can all encourage individuals to pursue extrinsic goals.

Both extrinsic and intrinsic goals contribute to well-being. Rijavec et al. (37) found that individuals with high levels of both intrinsic and extrinsic goals reported the highest levels of well-being. Cooper (53) posits that optimal well-being is achieved when intrinsic and extrinsic goals are harmoniously aligned. Therefore, we conclude that extrinsic goals do not invariably undermine well-being. The detrimental effect of extrinsic goals on well-being, as proposed in Goal Content Theory, does not hold in China. In a society deeply influenced by collectivist culture, extrinsic goals signify personal competence, serve as crucial means to achieve intrinsic goals, and are important factors in enhancing well-being. Research results extends and refines the Goal Content Theory.

### The mediation effect of extrinsic goals in Model 3

Unlike Model 1, 2, and 4, in Model 3, extrinsic goals, like intrinsic goals, also play a mediating role, rather than having a suppressing effect. By comparing the four models, it is found that this difference occurs because there are differences in the path through which extrinsic goals affect family happiness. Extrinsic goals negatively predict the outcome of family happiness, which is consistent with Goal Content Theory, but in contrast with the results of Model 1, 2, and 4. In Model 3, this path is negative, but in Models 1, 2, and 4, this path is positive. That is, extrinsic goals negatively affect the dimension of family happiness, but positively influence the overall good life experience and its two dimensions—national stability and personal richness. The influences of extrinsic goals on the three dimensions of a good life experience varies, indicating that Goal Content Theory is not completely inapplicable in the Chinese context.

This result can also explain why the results of the impact of extrinsic goals on well-being in previous studies are different (29, 42). We believe one of the reasons is that the impact of extrinsic goals on different dimensions of well-being is different, and the different dimensions’ proportions will impact the total effect. This provides a thought for cross-cultural research, that is, the differences in extrinsic goals may be due to different understandings of well-being in different cultures, or differences in the impact of extrinsic goals on different dimensions of subjective well-being.

### Policy recommendation

A good life is a shared pursuit and aspiration of the vast majority of Chinese people. On the one hand, death anxiety and neutral acceptance have distinct impacts on individuals’ good life experience. It is essential to actively guide the public to develop a scientific and rational understanding of death. This will help prevent their attitudes from being confined to instinctive anxiety and encourage individuals to approach life with optimism and positivity. On the other hand, intrinsic goals can enhance the positive impact of neutral acceptance on well-being, while extrinsic goals can mitigate the negative effects of death anxiety. Therefore, while striving to achieve extrinsic goals, it is crucial to guide the public toward pursuing intrinsic goals. This approach not only enables individuals to confront death more appropriately but also substantially enhances their overall well-being.

### Limitation and future prospect

First, the findings are based solely on cross-sectional survey data, which limits our ability to establish a causal relationship between death attitudes and the good life experience. Future research should utilize longitudinal data to explore this relationship more thoroughly. Second, to investigate the roles of intrinsic and extrinsic goals in death attitudes and good life experience, items that also encompass the attributes of self-transcendence or physical self were excluded. This approach controlled for the influence of confounding variables but may have limited the generalizability of the findings. Future research could further explore the roles of self-transcendence and physical self goals. Third, in this study we use good life satisfaction as the indicator of subjective well-being, but we have not compared it with the classic indicators of happiness. In the future, we can make comparisons among good life experience, life satisfaction, life vitality, positive and negative effects. Last, this study primarily focuses on exploring the influencing factors of subjective well-being from the psychological perspective. But well-being is influenced by multiple factors across various scales. Future research should emphasize cross-level integration to break down the barriers between different levels of factors, thereby investigating good life experience in multidimensional spaces and constructing a holistic model. According to the framework of Culturomics (54), subjective well-being research can be conducted integrated from the micro to the macro levels, examining the cross-levels variables and its interactions, and extracting well-being profiles from higher-order space, by the new method of deep neural network (DNN) or representation similarity analysis (RSA).

## Conclusion

1. This study examined the impact of Chinese individuals’ death attitudes on subjective well-being by good life experience. The findings reveal that death anxiety negatively predict the good life experience, whereas neutral acceptance positively predict it.
2. In the mechanism through which two types of death attitudes influence the good life experience, the intrinsic goal serves as a mediator in this relationship, while the extrinsic goal acts as a suppressor.
3. In the mechanism where the dimension of national stability serves as the outcome, the intrinsic goal acts as a mediator, while the extrinsic goal function as a suppressor, aligning with Model 1. However, in the mechanism where the dimension of family happiness serves as the outcome, both the intrinsic and extrinsic goals exhibit mediating effects.

## Data Availability

The data analyzed in this study are subject to specific licenses and restrictions. The dataset was collected through a large-scale survey platform and therefore does not include offline data. Requests to access the data should be directed to the corresponding author.
